# Risk factors for perioperative cerebral infarction in moyamoya disease: a meta-analysis

**DOI:** 10.3389/fneur.2025.1530137

**Published:** 2025-01-24

**Authors:** Jincan Wu, Shiju Li, Ruixin Liang, Yanxu Wang, Fangyuan Shi, Xiaoming Pan, Xinyi Chen

**Affiliations:** ^1^Fujian University of Traditional Chinese Medicine, Fujian, China; ^2^The Second Affiliated People's Hospital of Fujian University of Traditional Chinese Medicine, Fujian, China; ^3^People's Hospital Affiliated to Fujian University of Traditional Chinese Medicine, Fujian, China; ^4^Rehabilitation Hospital Affiliated to Fujian University of Traditional Chinese Medicine, Fujian, China

**Keywords:** risk factors, cerebral infarction, meta-analysis, moyamoya disease, perioperative

## Abstract

**Background:**

The present study explored the risk factors for cerebral infarction perioperative moyamoya disease by meta-analysis.

**Methods:**

The PubMed, Embase, Cochrane library, Web of science databases were searched for case–control/cohort studies on risk factors for the emergence of cerebral infarction perioperative moyamoya disease, the search was done from the database creation to June 1, 2024, and the data was analyzed by using stata15.0.

**Result:**

Ten retrospective cohort studies (*N* = 3,239) were included. Meta-analysis results suggested posterior cerebral artery involvement [OR = 2.62, 95%CI (1.36, 5.06)], preoperative magnetic resonance angiography [OR = 2.81, 95%CI (1.27, 6.22)], previous infarction [OR = 2.52, 95% CI (1.69, 3.75)] were risk factor for the development of cerebral infarction perioperative moyamoya disease.

**Conclusion:**

This study proves that posterior cerebral artery involvement and grade of preoperative magnetic resonance angiography is higher, and the previous infarction happened moyamoya disease a risk factor for cerebral infarction. Therefore, people with these risk factors should be intervened in advance to prevent the occurrence of perioperative cerebral infarction.

## Introduction

Moyamoya disease (MMD) is a chronic cerebrovascular disease with an unknown cause that involves progressive stenosis or occlusion of the ends of the internal carotid arteries (IC), anterior cerebral arteries (ACA), and the beginnings of the middle cerebral arteries (MCA) bilaterally, with the secondary formation of a smoky blood vessel network (1, 2). The age distribution of MMD has two peaks: around 10 years old and around 40 years old, but it is more common in adults (3). MMD can be categorized into ischemic and hemorrhagic types, with ischemic type being the most common. The main treatment means for this disease is intracranial and extracranial revascularization, which mainly includes direct bypass revascularization, indirect bypass revascularization and the combination of the two (4, 5). Bypass surgery can rapidly increase the cerebral blood flow in the anastomotic blood vessel supply area, improve the symptoms of cerebral ischemia, and reduce the probability of cerebral hemorrhage caused by rupture of collateral bypass vessels (6), which is significantly better than other treatment modalities in preventing cerebral infarction or cerebral hemorrhage, and previous studies have pointed out that the efficacy of direct bypass surgery or direct combined with indirect bypass surgery (referred to as combined bypass surgery) for patients with MMD is better compared with indirect bypass surgery (7, 8), so it has become the main treatment method for MMD. Therefore, it has become the preferred treatment option for MMD. Some studies (9–11) have pointed out that the most common complication after bypass surgery in MMD patients is cerebral infarction, the incidence of which ranges from 1.7 to 13.0%, which seriously affects the quality of life of the patients after the operation, and the personalized perioperative blood pressure management can effectively reduce the incidence and severity of cerebral infarction in the patients after the operation (12). Study (13) have suggested that the posterior cerebral artery increases the risk of cerebral palsy in patients with MMD, other (14) has found the opposite conclusion, the risk factors of perioperative cerebral infarction in Moyamoya disease are still controversial (15), therefore, this study aims to explore the risk factors of perioperative cerebral infarction in Moyamoya disease by meta-analysis, and to provide guidance for the improvement of the prognosis and quality of life of these patients.

## Materials and methods

### Literature retrieval

Two authors searched PubMed, Embase, Cochrane library, and web of science databases from database creation to June 1, 2024, with the search terms cerebral Infarction, risk factors, and moyamoya disease, and the specific search strategy is described in Supplementary Table S1.

### Literature selection

Inclusion criteria: adults who met the diagnostic criteria for moyamoya disease, exposure factor was perioperative cerebral infarction, primary outcome indicator was multivariate risk factors, and study type was case–control study or case–control study.

Exclusion criteria: duplicate articles, protocols, animal experiments, reviews, inaccessible full text, articles without usable data.

### Data extraction

Two authors rigorously selected the literature based upon predetermined inclusion and exclusion criteria. In case of any disagreement, they resolved it through discussion or sought the opinion of a third party to negotiate and reach consensus. Information extracted from the included studies included the following key details: study, year, country, study design, sample size, cerebral infarction, gender (M/F), mean age, regression model.

### Quality evaluation

The Newcastle-Ottawa Scale (NOS) (16) was used to evaluate case–control studies, including the selection of the study population (4 points), comparability between groups (2 points), and measurement of exposure factors or results (3 points). The total score of the scale is 9, with ≤4 indicating low quality, 5–6 indicating medium quality, and ≥ 7 indicating high quality. If the two researchers disagree on the evaluation process, they will discuss the decision or ask a third party to decide.

### Statistical analysis

The data were statistically analyzed using Stata 15.0 software. The OR (Odds ratio) and 95% CI (confidence interval) of the risk factors included in the articles were extracted. Heterogeneity test (Q test) and *I*^2^ statistic were used to select the appropriate model to calculate the combined OR. If *I*^2^ ≥ 50%, a random effects model was used; if I^2^ ≤ 50%, a fixed effects model was used. For *I*^2^ > 50%, we assessed the sensitivity of the literature using the leave-one-out method. In addition, we performed publication bias using the Egger test with the significance level set at *α* = 0.05. *p*-value <0.05 was considered statistically significant.

## Result

### Literature screening results

**Figure 1:**
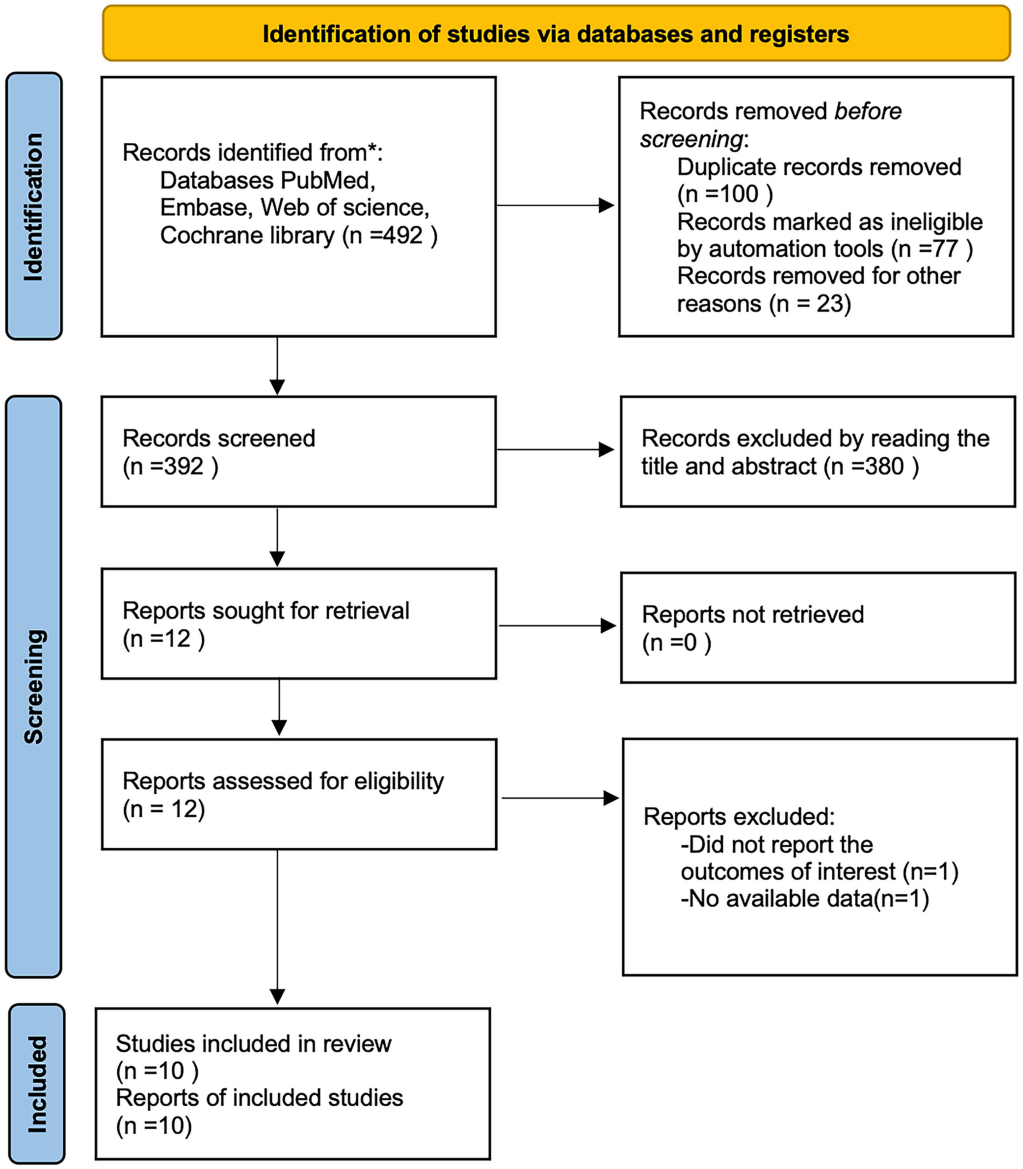
Literature search flow chart.

A total of 492 articles were obtained by searching PubMed, Embase, Cochrane library, and web of science databases, and 10 retrospective cohort studies (14, 17–25) were finally included by removing 100 duplicates, 380 articles by reading the title and abstract, and 2 articles by reading the full text (Figure 1), of which all 10 studies were cohort studies, all from Asian. The NOS scores of the included studies were 7 for 4 articles (17, 20, 24, 25), 8 for 3 articles (18, 21, 22), and 9 for 3 studies (14, 19, 23). The basic characteristics of the included studies are shown in Table 1.

**Table 1:** Basic features of included literatures.

Study	Year	Country	Study design	Sample size	Cerebral infarction	Gender (M/F)	Mean age	Regression model	NOS scores
Araki	2022	Japan	Cohort study	39	7	NR	3.6	logistic Regression	7
Choi	2020	Korea	Cohort study	1,241	63	551/690	3.7	logistic Regression	8
Guo	2023	China	Cohort study	160	60	79/81	2.17	logistic Regression	9
Hao	2024	China	Cohort study	308	36	152/156	8.5	logistic Regression	7
Hayashi	2021	Japan	Cohort study	120	54	53/67	6.7	logistic Regression	8
Li	2020	China	Cohort study	312	52	149/163	39.18	logistic Regression	8
Park	2016	Korea	Cohort study	194	44	58/136	37.2	logistic Regression	9
Qian	2020	China	Cohort study	250	31	108/142	30	logistic Regression	9
Wang	2022	China	Cohort study	890	46	432/289	38	logistic Regression	7
Yu	2023	China	Cohort study	418	49	198/220	38.3	logistic Regression	7

M/F, Male/female; NOS, Newcastle-Ottawa Scale.

### Meta-analysis results

#### Age

**Figure 2:**
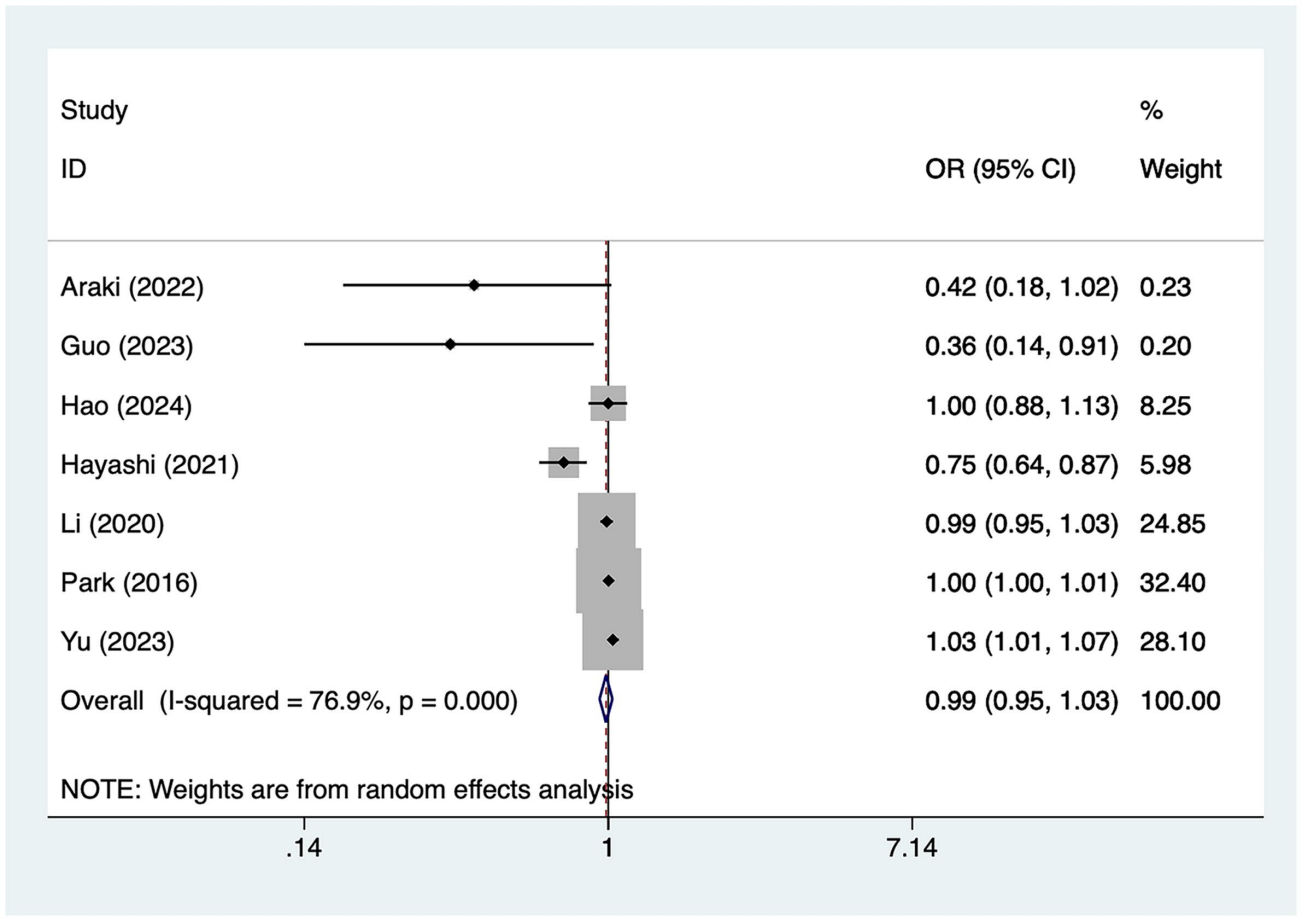
Forest plot of age meta-analysis.

A total of seven studies mentioned age, in which heterogeneity was tested (*I*^2^ = 76.9%, *p* = 0.001) and therefore analyzed using a random-effects model, and the results of the analysis (Figure 2) suggested that age was not a risk factor for the development of cerebral infarction perioperative moyamoya disease [OR = 0.99, 95% CI (0.95, 1.03), *p* = 0.14].

#### Posterior cerebral artery involvement

**Figure 3:**
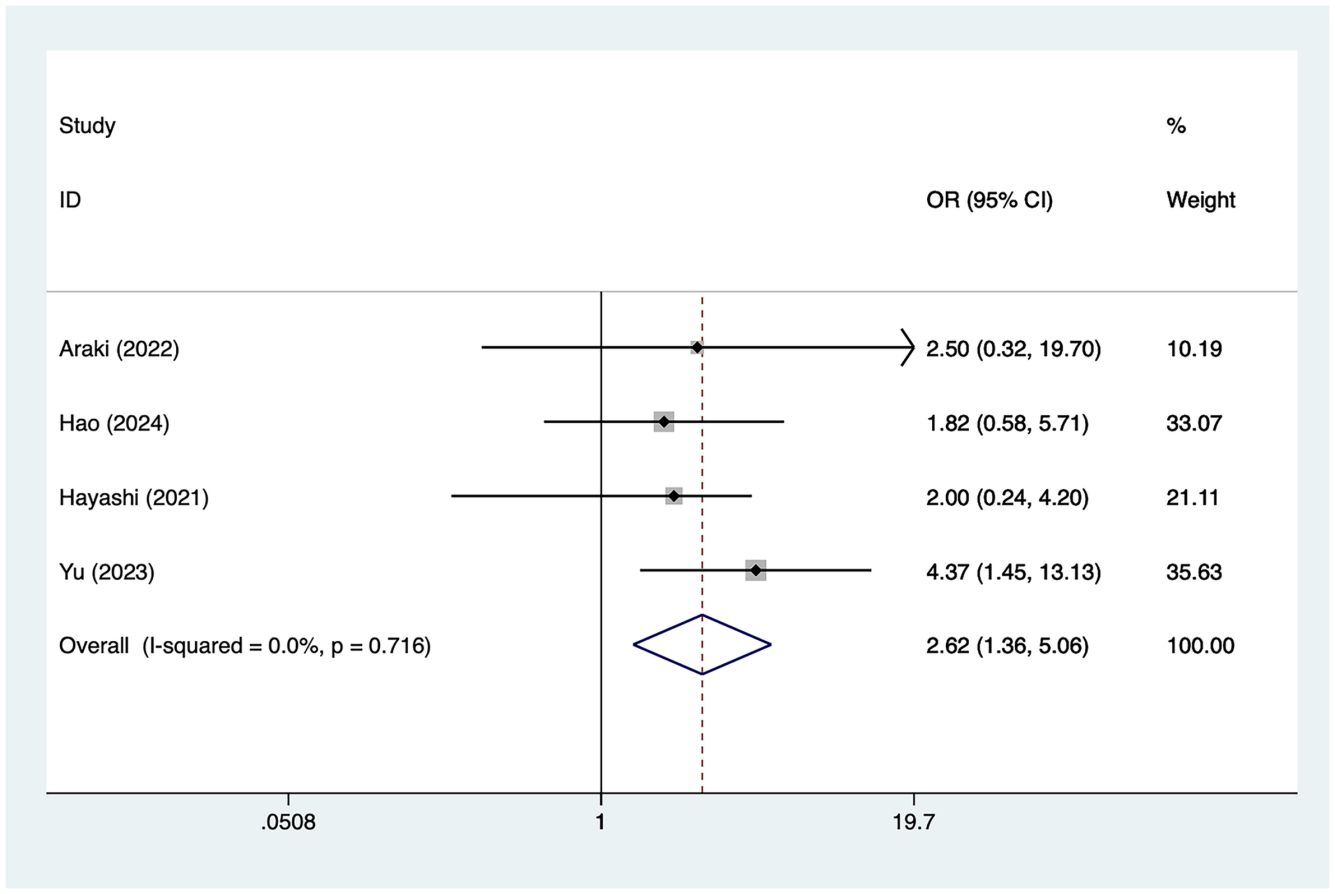
Forest plot of posterior cerebral artery involvement meta-analysis.

A total of three studies mentioned posterior cerebral artery involvement, in which heterogeneity was tested (*I*^2^ = 0.0%, *p* = 0.716) and therefore analyzed using a fixed-effects model, and the results of the analysis (Figure 3) suggested that posterior cerebral artery involvement was a risk factor for the development of cerebral infarction perioperative moyamoya disease [OR = 2.62, 95% CI (1.36, 5.06), *p* = 0.002].

#### Preoperative MRA grade

**Figure 4:**
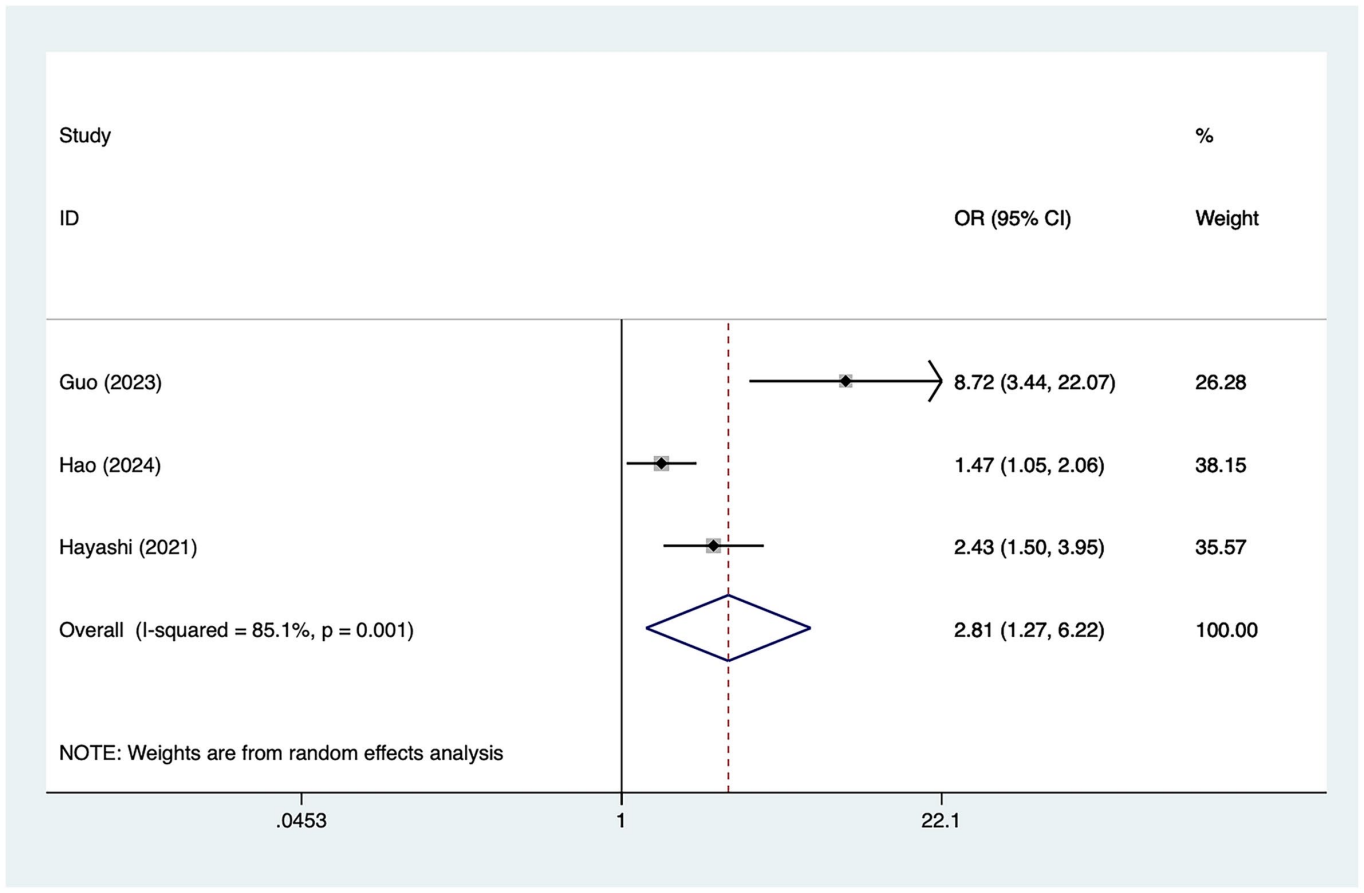
Forest plot of preoperative MRA grade meta-analysis.

A total of five studies mentioned preoperative magnetic resonance angiography (MRA) grade, in which heterogeneity was tested (*I*^2^ = 85.1%, *p* = 0.001) and therefore analyzed using a random-effects model, and the results of the analysis (Figure 4) suggested that preoperative MRA grade was a risk factor for the development of cerebral infarction perioperative moyamoya disease [OR = 2.81, 95% CI (1.27, 6.22), *p* = 0.003].

#### Previous infarction

**Figure 5:**
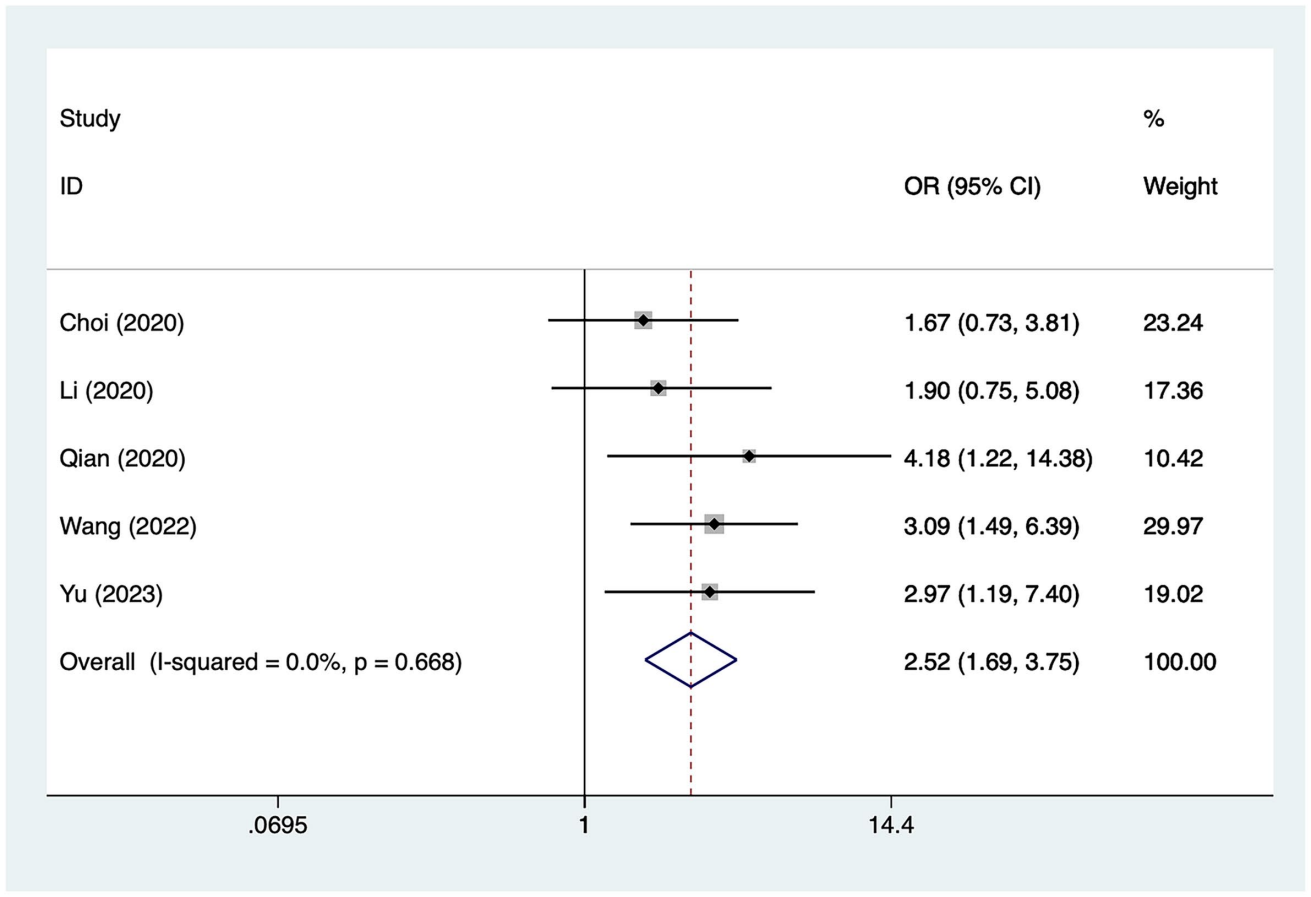
Forest plot of previous infarction meta-analysis.

A total of five studies mentioned previous infarction, in which heterogeneity was tested (*I*^2^ = 0.0%, *p* = 0.668) and therefore analyzed using a fixed-effects model, and the results of the analysis (Figure 5) suggested that previous infarction was a risk factor for the development of cerebral infarction perioperative moyamoya disease [OR = 2.52, 95% CI (1.69, 3.75), *p* = 0.01].

#### Female

**Figure 6:**
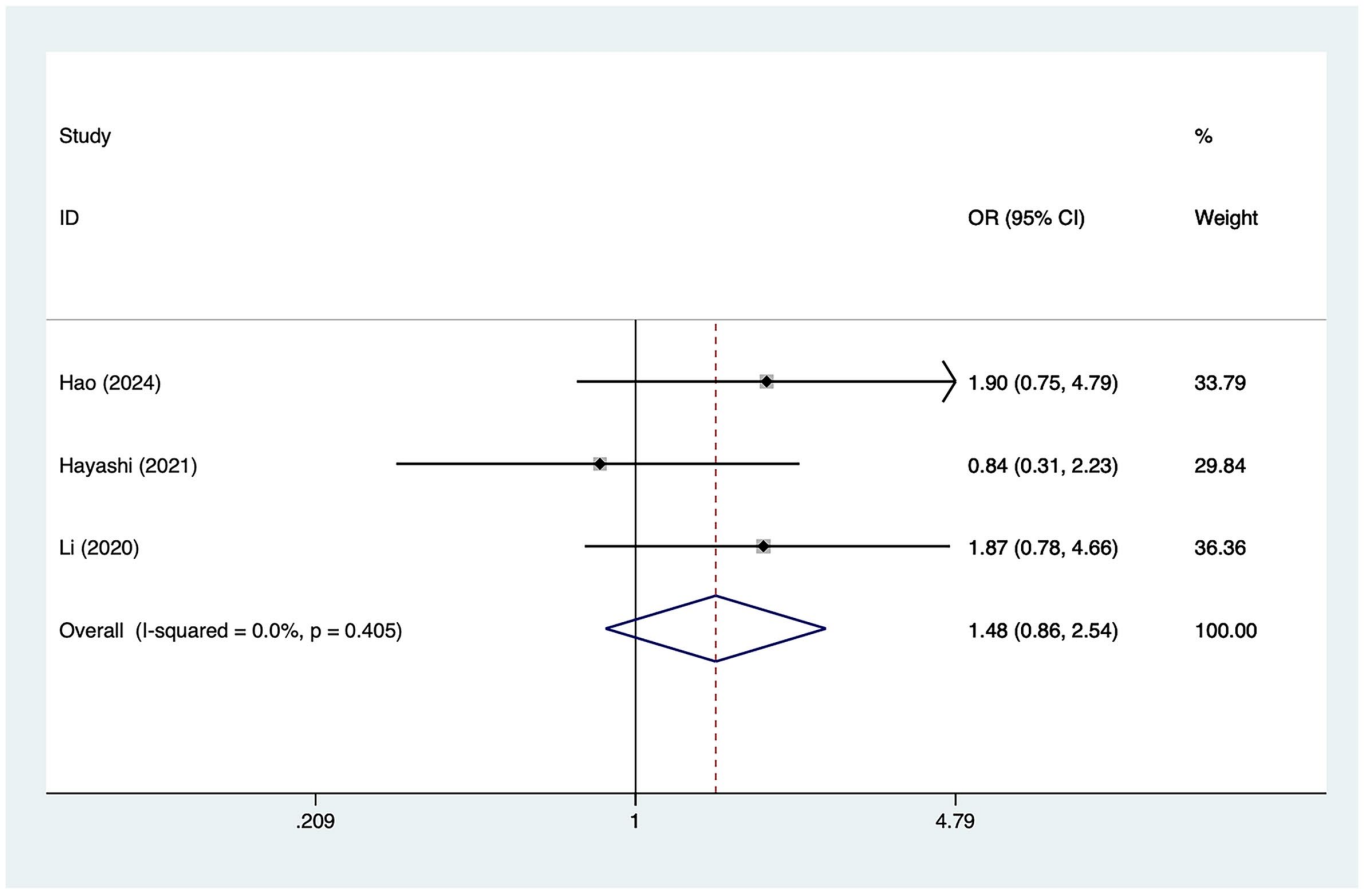
Forest plot of gender meta-analysis.

A total of three studies mentioned female, in which heterogeneity was tested (*I*^2^ = 0.0%, *p* = 0.405) and therefore analyzed using a fixed-effects model, and the results of the analysis (Figure 6) suggested that female was not a risk factor for the development of cerebral infarction perioperative moyamoya disease [OR = 1.48, 95% CI (0.86, 2.54), *p* = 0.53].

#### Hypertension

**Figure 7:**
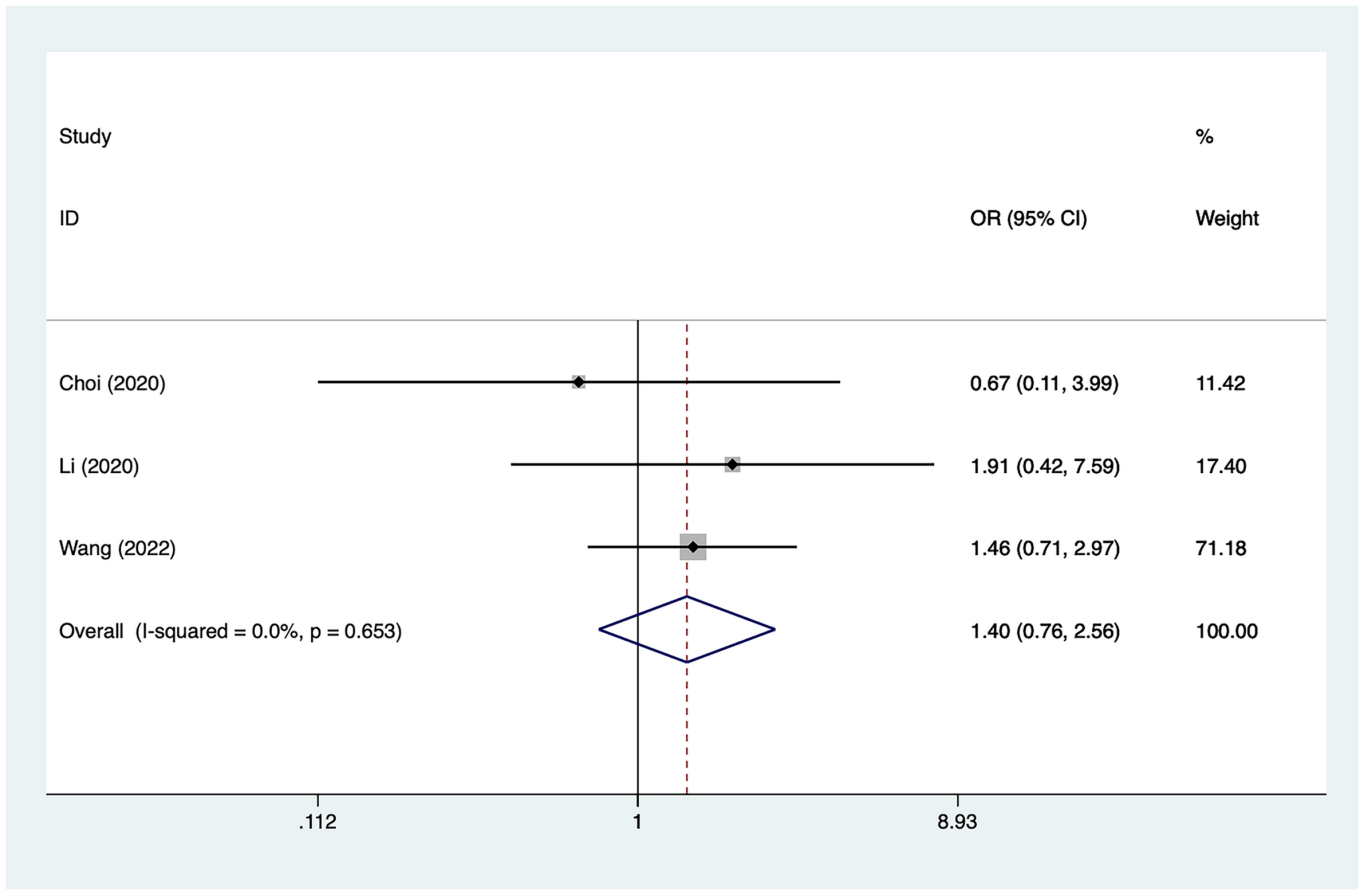
Forest plot of hypertension meta-analysis.

A total of three studies mentioned hypertension, in which heterogeneity was tested (*I*^2^ = 0.0%, *p* = 0.653) and therefore analyzed using a fixed-effects model, and the results of the analysis (Figure 7) suggested that hypertension was not a risk factor for the development of cerebral infarction perioperative moyamoya disease [OR = 1.40, 95% CI (0.76, 2.56), *p* = 0.12].

#### Publication bias

The included studies were analyzed for publication bias using Egger’s test, which suggested the possibility of publication bias for age (*p* = 0.51), posterior cerebral artery involvement (*p* = 0.13), preoperative magnetic resonance angiography (*p* = 0.45), previous infarction (*p* = 0.24), gender (*p* = 0.06), hypertension (*p* = 0.07), previous infarction(*p* = 0.24), female(*p* = 0.06), hypertension(*p* = 0.07) were less likely to have publication bias.

## Discussion

MMD blood flow reconstruction surgery is difficult to operate and has a high risk of bleeding; surgical stress, depth of anesthesia, operator proficiency, degree of refinement, and the physical quality of the patient may all have an impact on blood pressure (26, 27); in addition, arteries need to be blocked in the course of the surgery, which further reduces the cerebral perfusion; and it is difficult to control the fluctuation of the patient’s blood pressure in the course of the operation, making it difficult to ensure a stable perfusion of the brain tissue (28), and the patient’s incidence of cerebral infarction in the perioperative period is risk is significantly elevated (29, 30).

This study is the first to explore the risk factors for cerebral infarction after MMD, and it was found that MMD patients with posterior cerebral artery involvement are more likely to have cerebral infarction. First, stenosis or occlusion of the posterior cerebral artery occurs in the late Suzuki stage, and patients with this late stage have more severe intracranial stenosis, poorer cerebral perfusion, and more unstable hemodynamics (31, 32). Secondly, stenosis or occlusion of posterior cerebral artery involvement affects the compensatory capacity of collateral circulation in MMD patients, which is an important factor in maintaining cerebral perfusion in MMD patients, and furthermore, stenosis or occlusion of posterior cerebral artery involvement increases the risk of white blood clots (33). Furthermore, the relationship between posterior cerebral artery involvement stenosis or occlusion and revascularization is not only that posterior cerebral artery involvement stenosis leads to an increased risk of cerebral infarction in patients, but also that revascularization in patients with MMD may cause posterior cerebral artery involvement stenosis (34), therefore, for the population with posterior cerebral artery involvement, the progress of the disease should be closely monitored in clinical practice, and surgical intervention should be performed as early as possible to prevent the occurrence of cerebral infarction.

The study found that preoperative infarction is a risk factor for perioperative infarction in patients with MMD. Zhao et al. (35) proved that patients with preoperative ischemic manifestations had a significantly higher risk of perioperative ischemic complications through a study of 500 patients with MMD; Muraoka et al. (13) also proved that patients with preoperative cerebral infarction are more likely to have perioperative cerebral infarction than other, in fact, patients with preoperative ischemic attacks or cerebral infarction often had insufficient compensation of the side branches and extremely unstable cerebral hemodynamics, and mechanical damage to the compensated side branches during the surgical procedure and the sudden change of cerebral hemodynamics after revascularization were more likely to induce perioperative cerebral infarction (2, 36). In addition, transient ischemic attack can not only increase the occurrence of intravascular micro thrombosis but also increase the activity of serum inflammatory cytokines as well as related proteases, which further aggravates the occurrence of cerebral infarction (37), for MMD patients with preoperative infarction, life and exercise therapy should be carried out as early as possible to prevent postoperative cerebral infarction (38).

The current study found that higher Preoperative MRA grading was a risk factor for perioperative cerebral infarction in patients with MMD. The higher MRA stage on the non-operative side implies that the stenosis and occlusion of blood vessels on the non-operative side are more severe, and the ability of the non-operative side to compensate to the operative side is reduced, which is prone to cause perioperative cerebral infarction (39). Zhao et al. (35) showed that advanced MRA stage is a risk factor for ischemic complications in adult patients, therefore, for patients with higher grade of MRA before operation, early intervention and physical intervention should be performed to prevent the occurrence of cerebral infarction after operation (40).

The study still has the following limitations: first, fewer studies were included and the cut included studies were from Asia, which may affect the extrapolation of our conclusions; second, a part of the study population belonged to children and a part of the study population belonged to adults, which may be the source of our heterogeneity; third, some of the studies had a greater degree of heterogeneity but the test for heterogeneity was not able to find out the source of the heterogeneity.

## Conclusion

The present study demonstrated that posterior cerebral artery involvement, higher preoperative magnetic resonance angiography grading, and previous infarction are risk factors for the development of cerebral infarction perioperative moyamoya disease. Therefore, people with a combination of these risk factors should be intervened in advance to prevent perioperative cerebral infarction. However, since study limitations exist, In the future, we hope to have more studies with different regions, multi-centers and large samples to support our views.

## Data Availability

The original contributions presented in the study are included in the article/Supplementary material, further inquiries can be directed to the corresponding authors.
