# Supplementary material for: Risk factors for perioperative cerebral infarction in moyamoya disease: a meta-analysis
Source: Front Neurol. 2025 Jan 24;16:1530137. doi: 10.3389/fneur.2025.1530137 (PMC11802441; doi:10.3389/fneur.2025.1530137)
Supplement: Supplementary file 1 [file Data_Sheet_1.docx]

Table S1 Search strategy

((("Cerebral Infarction"[Mesh]) OR ((((((((((((((((((((((((((Cerebral Infarction[Title/Abstract]) OR (Cerebral Infarctions[Title/Abstract])) OR (Infarctions, Cerebral[Title/Abstract])) OR (Infarction, Cerebral[Title/Abstract])) OR (Cerebral Infarct[Title/Abstract])) OR (Cerebral Infarcts[Title/Abstract])) OR (Infarct, Cerebral[Title/Abstract])) OR (Infarcts, Cerebral[Title/Abstract])) OR (Cerebral Infarction, Left Hemisphere[Title/Abstract])) OR (Left Hemisphere, Infarction, Cerebral[Title/Abstract])) OR (Infarction, Left Hemisphere, Cerebral[Title/Abstract])) OR (Left Hemisphere, Cerebral Infarction[Title/Abstract])) OR (Cerebral, Left Hemisphere, Infarction[Title/Abstract])) OR (Infarction, Cerebral, Left Hemisphere[Title/Abstract])) OR (Subcortical Infarction[Title/Abstract])) OR (Infarction, Subcortical[Title/Abstract])) OR (Infarctions, Subcortical[Title/Abstract])) OR (Subcortical Infarctions[Title/Abstract])) OR (Posterior Choroidal Artery Infarction[Title/Abstract])) OR (Anterior Choroidal Artery Infarction[Title/Abstract])) OR (Cerebral Infarction, Right Hemisphere[Title/Abstract])) OR (Right Hemisphere, Cerebral Infarction[Title/Abstract])) OR (Infarction, Right Hemisphere, Cerebral[Title/Abstract])) OR (Right Hemisphere, Infarction, Cerebral[Title/Abstract])) OR (Cerebral, Right Hemisphere, Infarction[Title/Abstract])) OR (Infarction, Cerebral, Right Hemisphere[Title/Abstract]))) AND (("Risk Factors"[Mesh]) OR (((((((((((((((((((Risk Factors[Title/Abstract]) OR (Factor, Risk[Title/Abstract])) OR (Risk Factor[Title/Abstract])) OR (Social Risk Factors[Title/Abstract])) OR (Factor, Social Risk[Title/Abstract])) OR (Factors, Social Risk[Title/Abstract])) OR (Risk Factor, Social[Title/Abstract])) OR (Risk Factors, Social[Title/Abstract])) OR (Social Risk Factor[Title/Abstract])) OR (Health Correlates[Title/Abstract])) OR (Correlates, Health[Title/Abstract])) OR (Population at Risk[Title/Abstract])) OR (Populations at Risk[Title/Abstract])) OR (Risk Scores[Title/Abstract])) OR (Risk Score[Title/Abstract])) OR (Score, Risk[Title/Abstract])) OR (Risk Factor Scores[Title/Abstract])) OR (Risk Factor Score[Title/Abstract])) OR (Score, Risk Factor[Title/Abstract])))) AND (("Moyamoya Disease"[Mesh]) OR ((((((((((((((((((Moyamoya Disease[Title/Abstract]) OR (Progressive Intracranial Occlusive Arteropathy (Moyamoya[Title/Abstract]))) OR (Moyamoya Syndrome[Title/Abstract])) OR (Moya-Moya Disease[Title/Abstract])) OR (Disease, Moya-Moya[Title/Abstract])) OR (Moya Moya Disease[Title/Abstract])) OR (Cerebrovascular Moyamoya Disease[Title/Abstract])) OR (Moyamoya Disease, Primary[Title/Abstract])) OR (Disease, Primary Moyamoya[Title/Abstract])) OR (Moyamoya Diseases, Primary[Title/Abstract])) OR (Primary Moyamoya Disease[Title/Abstract])) OR (Primary Moyamoya Diseases[Title/Abstract])) OR (Primary Moyamoya Diseases[Title/Abstract])) OR (Moyamoya Disease, Classic[Title/Abstract])) OR (Classic Moyamoya Disease[Title/Abstract])) OR (Disease, Classic Moyamoya[Title/Abstract])) OR (Moyamoya Disease, Secondary[Title/Abstract])) OR (Secondary Moyamoya Disease[Title/Abstract])))
